# Primordial Biochemicals Within Coacervate-Like Droplets and the Origins of Life

**DOI:** 10.3390/v17020146

**Published:** 2025-01-23

**Authors:** George B. Stefano, Richard M. Kream

**Affiliations:** Department of Psychiatry, First Faculty of Medicine, Charles University and General University Hospital in Prague, Ke Karlovu 11, 120 00 Prague, Czech Republic; rmkream@sunynri.org

**Keywords:** coacervate droplets, environmental DNA, mitochondria, viruses, bacteria, evolution, reactive oxygen species

## Abstract

An organism is considered “alive” if it can grow, reproduce, respond to external stimuli, metabolize nutrients, and maintain stability. By this definition, both mitochondria and viruses exhibit the key characteristics of independent life. In addition to their capacity for self-replication under specifically defined conditions, both mitochondria and viruses can communicate via shared biochemical elements, alter cellular energy metabolism, and adapt to their local environment. To explain this phenomenon, we hypothesize that early viral prototype species evolved from ubiquitous environmental DNA and gained the capacity for self-replication within coacervate-like liquid droplets. The high mutation rates experienced in this environment streamlined their acquisition of standard genetic codes and adaptation to a diverse set of host environments. Similarly, mitochondria, eukaryotic intracellular organelles that generate energy and resolve oxygen toxicity, originally evolved from an infectious bacterial species and maintain their capacity for active functionality within the extracellular space. Thus, while mitochondria contribute profoundly to eukaryotic cellular homeostasis, their capacity for freestanding existence may lead to functional disruptions over time, notably, the overproduction of reactive oxygen species, a phenomenon strongly linked to aging-related disorders. Overall, a more in-depth understanding of the full extent of the evolution of both viruses and mitochondria from primordial precursors may lead to novel insights and therapeutic strategies to address neurodegenerative processes and promote healthy aging.

## 1. Environmental DNA and the Origins of Life

The primordial origins of nitrogen fixation and chemical diversity highlight the critical importance of bioavailable nitrogenous compounds as prerequisites for the emergence of life on Earth [1]. Theories on the origins of life emphasize the importance of alkaline hydrothermal vents found on the ocean floor that existed approximately 4.1 billion years ago during the anoxic Hadean period. These vents acted as electrochemical flow reactors where mineral surfaces rich in iron and nickel sulfides catalyzed the reduction of carbon dioxide (CO_2_) by molecular hydrogen (H_2_), ultimately forming methane. Concurrently, the influx of atmospheric nitrate (NO_3_^−^) and nitrite (NO_2_^−^) species into prebiotic Hadean oceans facilitated the generation of ammonia (NH_3_), a crucial precursor for nitrogen-containing biomolecules (e.g., amino acids, purine and pyrimidine bases, and nucleotides, which are essential components of proteins and nucleic acids [1]). This chemical framework likely laid the foundation for the emergence of diverse life forms during the Archean Eon (4000–2500 million years ago). Based on this scenario, we hypothesize that nucleic acid concatenation and base pairing first emerged in this primordial environment and represented the initial steps toward the evolution of all life forms.

These early biomolecules most likely existed in the primordial environment in a free-floating, non-membrane-bound state. Notably, environmental DNA (eDNA), which today refers to DNA fragments found in the extracellular spaces and environment, most likely played a significant role in these early precellular evolutionary processes. While current research on eDNA focuses primarily on its utility in environmental monitoring, its potential contributions and role as a dynamic participant in biological communication during early evolution most certainly warrant further consideration [2,3,4]. As an informational reservoir, eDNA/RNA may have contributed to life-originating processes by facilitating molecular interactions, including those that promoted stereospecific conformational matching, a mechanism through which molecules evolved toward greater stability and facilitated self-replication in specific environments. The genetic fragments that combined with one another and survived in these unique macromolecular microenvironments probably gave rise to both enveloped and non-enveloped virus species. We also surmise that various features of the primordial environment provided an advantageous thermodynamic setting that permitted this to occur.

Interestingly, independent, cell-free eDNA persists in many modern environments and across all domains of life. Interestingly, SARS-CoV-2 genetic material shed in human feces was subsequently detected in wastewater, thereby underscoring the environmental persistence of these genetic elements [2,3,4]. Here, we hypothesize that eDNA may contribute to the emergence of new viruses and provide molecular support for the ongoing evolution of all life on Earth.

## 2. Environmental DNA and the Emergence of Viruses

In contrast to the progressive and regressive hypotheses on the origin of viruses, which are both based on the assumption that cells predated viruses [5,6], Koonin and Martin suggest that viruses most likely emerged first because of their intrinsic capacity for replication [6,7]. According to this hypothesis, viruses may have originated as self-replicating entities from genetic fragments circulating in the environment in a primordial, precellular world (like eDNA). Over time, these replicative units increased in complexity, developing adaptive strategies for survival, environmental stability, and efficient mechanisms for DNA and/or RNA replication. This emerging complexity may have led to the formation of protective viral “envelopes” and, eventually, the emergence of prokaryotic cells. The long shared evolutionary history of viruses and prokaryotic (bacterial) cells and their many intricate interactions during reproduction support the plausibility of this hypothesis.

Interactions between eDNA and existing viruses may also provide the latter with several selective advantages. For example, eDNA fragments with structures or sequences that are similar to those found in viruses may enhance viral infectivity by facilitating selective binding to host cells. The eDNA environmental reservoir might also serve as a substrate for genetic recombination, enabling viruses to acquire genetic material from their surroundings and evolve into new strains or variants. Such processes not only influence viral evolution but may also have an immediate impact on host–virus interactions. Furthermore, the detection of SARS-CoV-2 DNA in wastewater demonstrates how eDNA might be used to monitor the prevalence of a given virus in a specific region or population.

## 3. Environmental DNA and the Evolution of Bacteria

A given pool of eDNA may also contain genetic material from bacteria, including sequences that may facilitate interactions within and between bacterial populations in the environment [8,9]. For example, eDNA may act as a scaffold for bacterial attachment and biofilm formation. Bacteria may also acquire new genetic material from eDNA that promotes their adaptation and evolution, including the spread of antibiotic resistance genes. Additionally, eDNA represents a source of nutrients that bacteria can metabolize for growth and survival. These findings highlight the multifaceted role of eDNA as a dynamic participant in both ancient and contemporary biological systems, with potential contributions to molecular evolution, microbial ecology, and the broader interplay of life forms and their environments.

Mitochondria, the evolutionary progeny of a free bacterial species, were not considered “alive” by conventional definitions because it was believed that they could not survive independently of their host cell. However, mitochondria possess their own DNA and the ability to replicate autonomously within the host [10,11]. Intriguingly, mitochondria emerged from an ancestral bacterial species involved in an intracellular infection of an early eukaryotic cell. This “symbiotic” relationship likely persisted because the invading bacterium provided a critical advantage by detoxifying oxygen, emerging from photosynthesis, and simultaneously enhancing cellular energy production [12,13]. In contrast to the interpretations of earlier experiments, recent work has revealed the presence of functioning mitochondria in cell-free extracellular spaces, demonstrating their impendence along with a role in supporting sensory functions [14,15].

As might be anticipated, pathogenic bacteria can target eukaryotic mitochondria and disrupt cellular energy metabolism as a strategy to enhance their survival [16,17]. Viral interactions with bacteria serve similar purposes, thereby emphasizing their significance in microbial survival strategies. These interactions may represent evolutionarily conserved competitive mechanisms that parallel those observed among bacterial populations. The resulting alterations in energy dynamics, particularly those that rely on oxygen availability, will most likely have a critical impact on key cellular processes. This may partially explain at least in part how antibiotics, which may target eukaryotic mitochondria in addition to freestanding bacteria, may influence cognitive function [18,19], leading to confusion, delirium, encephalopathy, and impaired concentration [20] as well as depression and autism [19].

With some notable exceptions [21], animal mitochondrial DNA (mtDNA) is devoid of introns. Furthermore, recent research has demonstrated that fragments of mtDNA can integrate into the nuclear genome, forming what are known as nuclear mitochondrial DNA segments (NUMTs) [22]. This discovery highlights the dynamic interaction between mitochondria and their host cells as well as the strength of mitochondrial influences. Intriguingly, these NUMTs have been associated with negative effects on the host organism; the results of this study revealed that NUMTs develop spontaneously and identified a link between cortical brain somatic NUMTs and human longevity [23].

**Figure 1:**
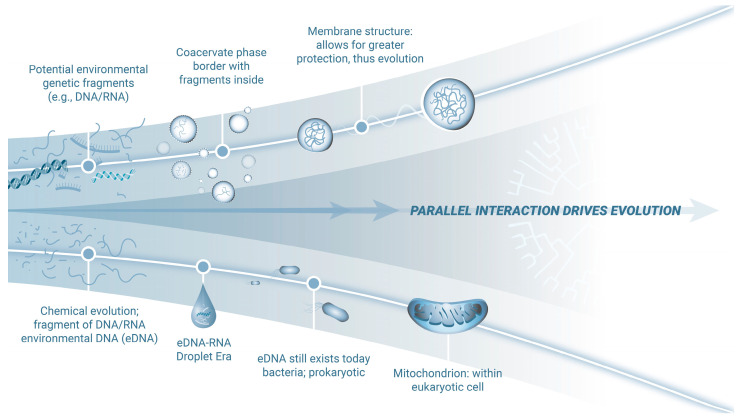
Impact of eDNA on the evolution and adaptation of mitochondria. Primordial eDNA-RNA likely formed via a thermodynamically favorable environment providing for relatively stable coacervate-like droplets. In this environment, genetic fragments would concentrate, interact, and facilitate novel base pairing in relative protection. Evidence suggests these fragments persist today, detectable in wastewater, intracellular and extracellular spaces, and mitochondria [22]. Additionally, bacterial and viral genetic material is present in human introns. Over time, in the primordial environment, these coacervate-like droplets evolved stable membranes, offering enhanced protection for genetic processes to progress strategically. Crucially, the reciprocal exchange of information with mitochondria, descendants of bacteria, likely enabled flexible, high-yield energy processes while mitigating the toxicity of photosynthesis-derived oxygen, offering a significant survival advantage. Thus, mitochondria became just one of the successful end products of this evolutionary process.

Collectively, these phenomena provide evidence of the nature of early evolutionary constraints and their persistence in current biological systems (Figure 1).

## 4. Facilitating Genetic Evolution: The Droplet Era

The microfluidic synthesis of membrane-free aqueous coacervate droplets offers an innovative framework for investigating the emergence of the primitive cell-free and cell-like structures that emerged during the early periods of biological evolution. This comparatively slow evolutionary process links eDNA (i.e., nucleic acid fragments in the environment) to prototype cell-like structures. Coacervate droplets are dense collections of biomolecules, including proteins and nucleic acids that undergo phase separation from the surrounding aqueous medium, resulting in the formation of distinct, membrane-free compartments that mimic key features of protocellular organization [24,25]. The nature and composition of coacervates can be influenced by environmental conditions, including temperature, pH, ionic strength, charge density, and the molecular weight of their constituent polyelectrolytes, among other factors [26]. It is speculated that these structures play a pivotal role in the evolutionary trajectory, leading to the formation of biological structures that contribute to the controlled microfluidic environment. These droplets are stabilized and maintained, and provide an excellent experimental model that can be used to explore how primitive cells may have evolved. Over time, the physicochemical conditions within the droplets could drive the self-assembly of lipid molecules, ultimately forming lipid bilayers and membranes [24,25]. This transition represents a critical milestone in the origin of life, as it facilitated the encapsulation of biochemical reactions and the creation of discrete microenvironments that are a prerequisite for the emergence of complex cellular processes.

Van Swaay and colleagues demonstrated that the size distribution and stability of coacervate droplets can be precisely controlled through microfluidic techniques, thus enabling their use as scalable, cell-like compartments [24]. As described by Agrawal and colleagues, the electrorheological properties of coacervates stem from their altered interfacial viscoelasticity and high polarizability in deionized water, which are characteristics that closely parallel those of living cells [25]. It can be further hypothesized that these microfluidic droplet systems facilitate the exchange of genetic information through several mechanisms that are similar to those used in biological processes. Droplet fusion permits multiple droplets to merge and mix their genetic contents, while diffusion through permeable interfaces facilitates the transfer of small DNA or RNA fragments between droplets [24,25]. These droplets can localize various genetic materials, for example, plasmids or viral genomes, and serve as “microreactors” that promote genetic processes, including nucleic acid replication or gene transcription. Given these findings, membrane-free coacervate droplets represent a compelling model that can be used to unravel the molecular and biophysical principles underlying the evolution of cellular membranes and provide a survival strategy for DNA/RNA together with the conservation and development of new information. By offering insights into the evolutionary processes that gave rise to the first cell-like structures, these systems provide a foundational platform for advancing our understanding of life’s origins and the evolution of cellular complexity over a long time period.

## 5. Evolution of Chemical Communication

The evolution of the prototype cell was guided by the development of complementary nucleotide sequences, which was and remains one of its sustaining features. These processes relied on stereospecific conformational matching, a process that facilitated precise interactions between signaling molecules, receptors, and enzymes across early non-cellular systems and later cell-based forms of life [27,28]. This foundation shared by viruses, bacteria, and eukaryotes highlights evolutionary continuity and facilitates mechanisms such as molecular mimicry [29]. The capacity for rapid mutation and shape recognition allowed pathogens to evolve to exploit “host” molecular structures. Thermodynamic stability provides additional support for these interactions and is a major factor driving evolutionary progress. One example of this important principle can be seen in the evolution of mitochondria, which evolved from a bacterial infective species into a permanent organelle in the eukaryotic cell.

As described, coacervates are dense, liquid-like droplets that form when their constituent macromolecules in solution separate into phases. These droplets can release chemical signals, for example, quorum-sensing molecules, inducers, or substrates, which are then detected by and trigger a biochemical response in a neighboring droplet that contains specific receptors or molecular sensors [24,25]. These interactions are similar to those reported for vesicles or liposomes, which are membrane-bound structures that can localize, contain, and transfer various molecules, including genetic material and proteins, in a eukaryotic environment. Interestingly, these vesicles can fuse with the droplet membrane and transfer their contents, thereby simulating the way cells take up extracellular vesicles or viruses and thus transfer genetic material and other biochemicals. Clearly, the essential elements of this ancient process remain, demonstrating their importance and success as well as a critical means for ongoing evolution.

Traditionally, nucleic acid molecules are not considered “alive”. However, if these molecules can undergo self-supported replication within a prototype system, they may qualify as living entities [30]. For example, consider the case of a droplet containing genetic material (e.g., plasmids or DNA fragments) or proteins (e.g., transcription factors) found in the vicinity of a recipient droplet. In response to an electric field or a mechanical force, these droplets can merge, a process that will lead to the direct transfer of the material from one droplet to another. Once inside, the genetic material from the donor droplet will have the opportunity to interact with cognate elements found in the recipient and trigger recombination and/or signaling cascades. This simple yet plausible mechanism mirrors our current understanding of the behavior of viruses, which have evolved over billions of years, most likely by using similar mechanisms before the emergence of true cell membranes. Collectively, these insights suggest that viruses may represent an ancient form of biological communication that emerged and persisted throughout evolution, representing a successful remnant of early evolutionary processes that developed in the precellular “droplet” era and involving newly developed forms of eDNA/RNA.

## 6. Conclusions

Taking all of this information into consideration, we hypothesize that both viruses and mitochondria can be considered living entities because of their capacity for self-replication and their independence in specific biological environments. Furthermore, their ongoing evolution provides many examples of complexity and adaptation that have taken place over billions of years; they may currently represent the most successful life forms on Earth. While modern-day eukaryotic cells would no longer exist in their absence, viruses and mitochondria can continue to evolve in the absence of eukaryotic architecture. In this sense, eukaryotic cells may be perceived as elements of a sophisticated protection strategy that has developed to support bacteria and viruses. Interestingly, mitochondria may represent a chronic infection that has been fine-tuned to function within the existing eukaryotic architecture that fails over time due to intrinsic and extrinsic influences (e.g., free radicals). Further research into the origins and evolution of viruses and mitochondria may ultimately lead to therapeutic strategies designed to address neurodegenerative processes and promote healthy aging.

As stated by the eminent 19th-century biologist, Charles Darwin, one does not need to be large, strong, or intelligent to survive, merely adaptable [31].
